# The optimal route of progesterone administration for luteal phase support in a frozen embryo transfer: a systematic review

**DOI:** 10.1007/s00404-022-06674-2

**Published:** 2022-08-09

**Authors:** Abdulla Almohammadi, Ainharan Raveendran, Mairead Black, Abha Maheshwari

**Affiliations:** 1 Department of Reproductive Medicine, Sidra Medicine Hospital, Doha, Qatar; 2 Department of Obstetrics and Gynaecology, Aberdeen Royal Infirmary, Aberdeen Maternity Hospital, Aberdeen, UK; 3 University of Aberdeen, Aberdeen Maternity Hospital, Aberdeen Centre for Women’s Health Research, Aberdeen, UK; 4 Aberdeen Fertility Centre, Aberdeen Maternity Hospital, Reproductive Medicine, Aberdeen, UK

**Keywords:** Luteal phase support, Frozen embryo transfer, Progesterone, Live birth, Miscarriage

## Abstract

**Objective:**

To investigate the optimal route of progesterone administration for luteal phase support in a frozen embryo transfer.

**Design:**

Systematic review.

**Patients:**

Women undergoing frozen embryo transfer (FET).

**Interventions:**

We conducted an extensive database search of Medline (PubMed), Embase, Web of Science, and Cochrane Trials Register using relevant keywords and their combinations to find randomized controlled trials (RCTs) comparing the routes (i.e., oral, vaginal, intramuscular) of progesterone administration for luteal phase support (LPS) in artificial FET.

**Main outcome measures:**

Clinical pregnancy, live birth, miscarriage.

**Results:**

Four RCTs with 3245 participants undergoing artificial endometrial preparation (EP) cycles during FET were found to be eligible. Four trials compared vaginal progesterone with intramuscular progesterone and two trials compared vaginal progesterone with oral progesterone. One study favored of vaginal versus oral progesterone for clinical pregnancy rates (RR 0.45, 95% CI 0.22–0.92) and other study favored intramuscular versus vaginal progesterone for clinical pregnancy rates (RR 1.46, 95% CI 1.21–1.76) and live birth rates (RR 1.62, 95% CI 1.28–2.05). Tabulation of overall evidence strength assessment showed low-quality evidence on the basis that for each outcome-comparison pair, there were deficiencies in either directness of outcome measurement or study quality.

**Conclusion:**

There was little consensus and evidence was heterogeneous on the optimal route of administration of progesterone for LPS during FET in artificial EP cycles. This warrants more trials, indirect comparisons, and network meta-analyses.

**PROPERO No:**

CRD42021251017.

## What does this study add to the clinical work

**Table:** 

We sought to evaluate the current evidence regarding the optimal route of progesterone administration for luteal phase support in women undergoing FET cycles.	

## Introduction

Infertility is a prevalent public health issue, affecting 15% couples of reproductive age worldwide [1]. It is on the rise with 48 million couples and 186 million individuals infertile all over the world [2]. It is a life crisis with damaging psychosocial consequences in the form of marital instability, violence, divorce, social exclusion, stigmatization, and suicidal ideations [3]. Infertility is considered a personal failure or tragedy across the world [4]. Therefore, scientific efforts have come up with different ways to treat infertility including fertility counseling, lifestyle modifications, drug or hormone therapy, surgical procedures and assisted reproductive technology (ART) [5]. Assisted reproductive technologies include in vitro fertilization (IVF), cryopreservation and intracytoplasmic sperm injection (ICSI) which manipulate with eggs or embryos and provide an effective infertility solution [6].

Advances in cryopreservation techniques have improved the survival of blastocyst stage embryos resulting in a significant rise in the frozen–thawed embryo transfers (FETs) [7–9]. The indications for FET are broad and need to be considered in the context of how ART is evolving. ART indications have expanded to include both male and female causes of infertility as well as non-infertility indications such as pre-implantation genetic diagnosis. FET being a laboratory technique is thus amenable to use across ART indications including tubal infertility, endometriosis, and male infertility. The prevention of ovarian hyperstimulation syndrome may be seen as a specific reason, though patient preference for timing of conception in any accepted indication is a key reason. Thus, FET is a rapidly increasing technique for deployed in ART [7–9]. However, failure of implantation is one of the major causes limiting its success. Progesterone produced by the corpus luteum supports the pregnancy in natural cycles playing a key role in endometrial preparation for implantation to take place successfully. In FETs, lack of progesterone due to the absence of corpus luteum requires external progesterone supplementation, which is also known to play a role in prevention of miscarriage [10]. Although the outcome of natural cycle FET and artificial cycle FET have equally effective pregnancy outcomes; however, artificial cycle FET has shown to be easier to monitor and plan a date with low cancelation rate [11].

FETs may suffer iatrogenic luteal phase defects in artificial endometrial preparation (EP) [12]. FETs necessitate the coordination of development of endometrium in synchrony with the embryo’s developmental stage for successful outcomes [13]. One approach is to modify the natural cycle by inducing ovulation with HCG [14]. In artificial EP cycles, where there is no corpus luteum, hormonal supplementation or progesterone replacement is deployed via various routes. Therefore, most FET cycles are Hormonal mediated for convenience of patients and planning in clinics [15]. However, the optimal treatment for luteal phase support (LPS) remains a recognized matter of debate [16].

Luteal phase support with progesterone is expected to play a fundamental role in maintaining early pregnancy, optimizing the outcomes of FET including ongoing pregnancy and live birth rate [17]. In FET treatment, clinically important serum level of progesterone in luteal phase is required to ensure adequate milieu for embryo implantation [18]. Various routes of progesterone supplementation used during LPS in FET have been evaluated in numerous individual studies showing different effects on pregnancy rates. Some of the previous reviews based on quality assessment tools assessing the optimal route of progesterone administration during luteal phase in FET treatment are old, have not included all the routes or have reported varied optimal routes [19–22].

In general, it has been suggested that while the efficacy of both oral and intramuscular progesterone is comparable to that of the vaginal route, the latter has better acceptance and tolerance. However, oral administration is more patient-friendly than the vaginal route [35, 36]. Hence, ideal route for LPS is yet to be determined, warranting a new evidence synthesis for considering the various progesterone administration routes together for their relative effectiveness. Therefore, we conducted this systematic review to collate the evidence from all published randomized controlled trials (RCTs) comparing progesterone administered via the oral, vaginal, and parenteral (subcutaneous or intramuscular) routes for LPS in artificial EP cycles during FET.

## Materials and methods

### Registration

A review was prospectively registered to identify, appraise, and summarize the evidence from RCTs examining the effects of progesterone given for LPS in artificial EP via oral, vaginal, and intramuscular means to determine the optimal route of progesterone administration in women undergoing FET (PROSPERO No. CRD42021251017). This systematic review followed the guidelines of PRISMA 2020 statement [23].

### Search strategy

In June 2021, we conducted an extensive search on the databases of Medline (PubMed), Embase, Web of Science, and Cochrane Trials Register using certain keywords and their combinations such as “LPS and FET (participants) and progesterone (intervention)”. No restrictions regarding year of publication or language of the article were applied. Update searches were conducted in May 2022. Randomized controlled trials comparing the routes (i.e., oral, vaginal, parenteral) of progesterone administration for LPS in artificial FET included in the review. Non-randomized studies and those including participants with natural or modified natural cycles were excluded from the review. Further, the registered RCTs in registries without published data were removed from the review. Duplicate citations were removed electronically from the combined electronic searches. Reference lists of included studies were also checked to find possible relevant citation that might have been missed by electronic searches.

### Study selection and quality assessment

Titles and abstracts of the articles were reviewed independently by two reviewers (Abdulla Almohammadi and Ainharan Raveendran) and the full texts of the potentially relevant articles were acquired. Full texts were then assessed independently by two reviewers (Abdulla Almohammadi and Ainharan Raveendran) for relevancy, and disagreements were settled by discussion with the third author (Dr. Mairead Black). The methodological quality was assessed using the current version of the Cochrane risk of bias tool [24]. It covered five domains of (1) randomization process or selection bias, (2) intended interventions or performance bias), (3) missing outcome data or attrition bias, (4) outcome assessment or measurement bias, and (5) selective reporting. Domain (4) concerning outcome measurement examined if the outcome was directly measured using established objective criteria with ultrasound for clinical pregnancy.

### Study outcomes

The outcomes included clinical pregnancy, live birth and early pregnancy loss, which were defined and measured according to published core outcome sets [25].

### Data extraction process

Two reviewers (Abdulla Almohammadi and Ainharan Raveendran) extracted the data on the variables related to study characteristics, participants, interventions, and outcomes. Any disagreements were settled by discussion with the third author (Dr. Mairead Black).

### Data synthesis and analysis

The characteristics of the studies including the different doses of the drug for each route were tabulated to describe the interventions. The study quality data were tabulated and plotted in a stacked bar chart. Numbers and percentages of events per intervention group were computed and tabulated, focusing on outcomes prospectively registered i.e., clinical pregnancy, live birth, and miscarriages. Results of individual studies were computed as relative risk (RR) with 95% confidence intervals (CI) and tabulated and grouped according to outcome-intervention pairs.

Evaluation of appropriateness of meta-analysis, statistical combination of individual studies into a single summary was planned, considering homogeneity of comparisons, outcomes, study quality and consistency of individual results. To qualitatively evaluate heterogeneity or inconsistency of results between studies, direction of point estimates of effects in individual studies were compared where more than one studies were available. If the individual point estimates were on both sides of the ‘no effect’ relative risk value 1.0, the results were indicative of both beneficial and harmful effects, so they were considered inconsistent qualitatively. Statistical tests for heterogeneity were not performed due to small number of studies available for reliable assessment.

Overall evidence strength was summarized in tables including study design, directness of outcome measurement, study quality assessment, inconsistency of results (heterogeneity of point estimates), and imprecision of individual effects (95% CIs including the relative risk value 1.0). Publication bias could not be assessed as there were too few studies for reliable assessment.

## Results

### Included studies

**Fig. 1:**
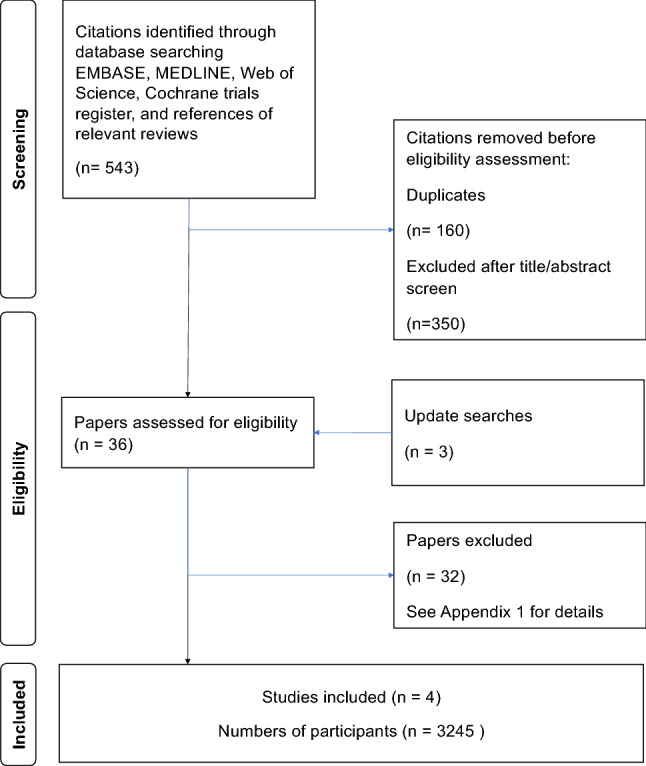
Study search and selection flow chart in the systematic review of progesterone supplementation for luteal phase support

The search yielded 543 citations in the original search. After removal of duplicates and irrelevant titles/abstracts, and addition of 3 citations captured in update searches, 36 studies were shortlisted for review of full-text articles. Four RCTs with 3245 participants undergoing artificial EP cycles during FET were found to be eligible [26–29] (Fig. 1). The excluded studies were either not randomized or included participants with modified natural FET cycles or were an interim analysis of a full report as listed in Appendix 1.

### Study characteristics

The included studies were recent conducted in Iran, USA, and China. Vaginal progesterone was used in all trials. Four trials compared vaginal progesterone with intramuscular progesterone and two trials compare vaginal progesterone with oral progesterone (Table 1). Two trials also evaluated combination of routes for progesterone replacement. The included RCTs used various doses and progesterone types.

**Table 1:** Characteristics of the studies included in the systematic review of progesterone supplementation for luteal phase support

Author, year	Country, sample size	Oral intervention route and dose	Vaginal intervention route and dose	Intramuscular intervention route and dose	Combination intervention route and dose
Rashidi et al. [26], 2016	Iran, *n* = 180	Oral dydrogesterone (40 mg daily)	Vaginal progesterone (800 mg per day)	Intramuscular (100 mg per day)	
Devine et al. [29], 2021	United States, *n* = 1125		Vaginal progesterone endometrin (400 mg daily)	Intramuscular progesterone (50 mg daily)	Vaginal progesterone endometrin (400 mg daily) and intramuscular progesterone (50 mg every 3rd day)
Zarei et al. [28], 2018	Iran, *n* = 440	Oral dydrogesterone (20 mg)	Vaginal progesterone (800 mg daily)		Oral dydrogesterone (20 mg) and gonadotropin releasing hormone analog (0.1 mg); and oral dydrogesterone (10 mg) and human chorionic gonadotrophin injection (1500 IU)
Wang et al. [27], 2015	China, *n* = 1500		Vaginal progesterone (90 mg)	Intramuscular progesterone (40 mg)	

### Study quality assessment

**Fig. 2:**
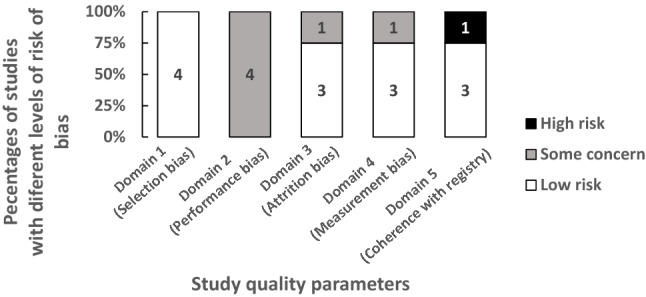
Study quality assessment in the systematic review of progesterone supplementation for luteal phase support (summarized using individual study quality data in Table 2)

Risk of bias was low concerning randomization process, but there were some concerns with respect to intended interventions, missing data, outcome measurement and selective reporting (Table 2, Fig. 2). With respect to directness of outcome measurement, clinical pregnancy was confirmed by ultrasound showing viable fetus or intrauterine gestational sac in three studies [26, 27, 29]. Clinical pregnancy confirmation was not reported in one study [28]. One study had deficiencies in four domains [28].

**Table 2:** Risk of bias assessment of individual studies included in the systematic review of progesterone supplementation for luteal phase support

Author, year	Domain 1: randomization process (selection bias)	Domain 2: intended interventions (performance bias)	Domain 3: missing outcome data (attrition bias)	Domain 4: outcome assessment (measurement bias)	Domain 5: selective reporting (coherence with registry)
Rashidi et al. [26], 2016	Low	Some concern	Low	Low	Low
Devine et al. [29], 2021	Low	Some concern	Low	Low	Low
Zarei et al. [28], 2018	Low	Some concern	Some concern	Some concern	High
Wang et al. [27], 2015	Low	Some concern	Low	Low	Low

### Synthesis of results

Table 3 gives the rates of clinical pregnancy, live birth, and miscarriages per intervention group per study. Devine et al. [29] gave data on live birth and miscarriage updating on the previously reported interim analysis. Zarei et al. [28] did not report live birth. It was not possible to determine which route had higher outcome rates. Only two studies showed statistically significant results. One study was in favor of vaginal versus oral progesterone for clinical pregnancy rates (RR 0.45, 95% CI 0.22–0.92) [28]; however, this study suffered methodological weaknesses (Tables 2 and 4). The other study was in favor of intramuscular versus vaginal progesterone for clinical pregnancy rates (RR 1.46, 95% CI 1.21–1.76) and live birth rates (RR 1.62, 95% CI 1.28–2.05) [29]; and, this study had methodological strength (Tables 2 and 4). For other outcome-comparison pairs, individual results were not statistically significant (Table 4). Tabulation of overall evidence strength assessment showed low-quality evidence on the basis that for each outcome-comparison pair, there were deficiencies in either directness of outcome measurement or study quality, i.e., concerns with respect to risk of bias, or precision, i.e., 95% CIs including the relative risk value 1.0 (Table 5).

**Table 3:** Results concerning the outcomes among studies included in the systematic review of progesterone supplementation for luteal phase support

Author, year	Outcome measure^$^	Oral intervention n/t (%)	Vaginal intervention n/t (%)	Intramuscular intervention n/t (%)
Rashidi et al. [26], 2016	Clinical pregnancy rate	22/60 (36.66%)	17/60 (28.33%)	23/60 (38.33%)
	Live birth rate	17/60 (26.66%)	16/60 (26.66%)	18/60 (30%)
	Early pregnancy loss (spontaneous abortion rate)^+^	2/22 (9.0%)	1/17 (5.88%)	2/23 (8.69%)
Devine et al. [29], 2021	Clinical pregnancy rate		86/231 (37%)	229/421 (54%)
	Live birth rate Early pregnancy loss (Clinical loss)		63/231 (27%) 23/86 (27%)	186/421 (44%) 43/229 (19%)
Zarei et al. [28], 2016	Clinical pregnancy rate	9/100 (9.0%)	20/100 (20%)	
	Ongoing pregnancy rate	9/100 (9.0%)	18/100 (18%)	
	Early pregnancy loss (miscarriage rate)^+^	5 (35.7%)	4 (18.1%)	
Wang et al. [27], 2015	Clinical pregnancy rate		289/721 (40.1%)	295/726 (40.6%)
	Live birth rate		235/721 (32.6%)	230/726 (31.7%)
	Early pregnancy loss (early abortion rate)		47/289 (16.3%)	54/295 (18.3%)

^$^Clinical pregnancy confirmed by ultrasound showing viable fetus at 6 weeks by Rashidi et al. [26], intrauterine gestation sac at 5 weeks by Devine et al. [29] and intrauterine gestation sac at 4–6 weeks by Wang et al. [27] after embryo transfer, but confirmation not reported by Zarei et al. [28]

*Data for other interventions listed in Table 1 not identified separately in the paper which reports an interim analysis;

^+^Reported data do not provide correct numerical denominators

**Table 4:** Individual effect sizes calculated as relative risk point estimate with 95% confidence interval (CI) for various comparisons-outcomes pairs amongst studies included in the systematic review of progesterone supplementation for luteal phase support

Outcome measure$	Author, year	Oral vs vaginal Relative risk (95% CI)	Intramuscular vs vaginal Relative risk (95% CI)	Intramuscular vs oral Relative risk (95% CI)
Clinical pregnancy rate	Rashidi et al. [26], 2016	1.29 (0.77–2.19)	1.35 (0.82–2.27)	1.35 (0.82–2.27)
	Devine et al. [29], 2021		1.46 (1.21–1.76)*	
	Zarei et al. [28], 2016	0.45 (0.22–0.92)*		
	Wang et al. [27], 2015		1.01 (0.89–1.15)	
Live birth rate	Rashidi et al. [26], 2016	1.06 (0.60–1.89)	1.13 (0.64–1.99)	1.06 (0.61–1.84)
	Devine et al. [29], 2021		1.62 (1.28–2.05)*	
	Zarei et al. [28], 2016	0.50 (0.24–1.04)		
	Wang et al. [27], 2015		0.97 (0.84–1.13)	
Early pregnancy loss +	Rashidi et al. [26], 2016	1.55 (0.22–11.3)	1.48 (0.21–10.8)	0.96 (0.18–5.1)
	Devine et al. [29], 2021		0.70 (0.45–1.09)	
	Zarei et al. [28], 2016			
	Wang et al. [27], 2015		1.13 (0.80–1.61)	

^$^Clinical pregnancy confirmed by ultrasound showing viable fetus at 6 weeks by Rashidi et al. [26], and intrauterine gestation sac at 4–6 weeks by Wang et al. [27] after embryo transfer, but confirmation not reported by Zarei et al. [28]

*Statically significant, *p* < 0.05

+ See Table 4 for variation in outcome measurement

**Table 5:** Overall evidence strength assessment in the systematic review of progesterone supplementation for luteal phase support

Route comparison and outcome	Study design	Directness of outcome measure	Study quality (risk of bias in Table 2)	Inconsistency of results (heterogeneity of point estimates)*	Imprecision of effects #	Publication bias (too few studies for assessment)
Oral vs. vaginal						
Pregnancy rate	RCT	Some indirectness $	Serious limitations	Inconsistent	Imprecise	Not assessed
Live birth	RCT	Direct	Serious limitations	Inconsistent	Imprecise	Not assessed
Early pregnancy loss	RCT	Some indirectness +	Some limitations	Not assessed	Imprecise	Not assessed
Intramuscular vs vaginal						
Pregnancy rate	RCT	Direct	Some limitations	Consistent	Imprecise	Not assessed
Live birth	RCT	Direct	Some limitations	Inconsistent	Imprecise	Not assessed
Early pregnancy loss	RCT	Some indirectness +	Some limitations	Consistent	Imprecise	Not assessed
Intramuscular vs oral						
Pregnancy rate	RCT	Direct	Serious limitations	Not assessed	Imprecise	Not assessed
Live birth	RCT	Direct	Serious limitations	Not assessed	Imprecise	Not assessed
Early pregnancy loss	RCT	Some indirectness +	Serious limitations	Not assessed	Imprecise	Not assessed

^$^Zarei et al. [28] did not report determination of outcome measure as shown in Table 3

+ Various measures used as shown in Table 3

*Individual point estimates of effect in opposite directions used to assess heterogeneity qualitatively when there were more than one studies in the outcome-comparison pairs as shown in Table 4

^#^Meta-analysis not used and only width of individual 95% confidence interval assessed with respect of overlapping of the ‘no effect’ relative risk value 1.0 as shown in Table 4

## Discussion

This focused systematic review aimed to investigate the relationship between route of progesterone supplementation and FET outcomes in artificial EP cycles. The quality of the included studies was diverse, predominantly moderate in risk of bias. A range of administration routes were covered in the included studies. There were a variety of doses and progesterone types were used making meta-analysis unsuitable. This heterogeneity is probably related to the different treatment policies among the countries and IVF units included in the review. Only one study (at high risk of bias) showed a statistically significant result in favor of vaginal versus oral progesterone for clinical pregnancy rates; for other outcome-comparison pairs, individual results were not statistically significant. Considering the risk of bias and imprecision of results, it was not possible to determine which route had the best outcome.

This systematic review followed a robust methodology to attempt to reduce the possibility of various forms of errors and biases. There was prospective registration and compliance with PRISMA guideline for reporting [23]. The published core outcome set for infertility research was used to select critical and important outcomes [25]. The global search without language and date restrictions yielded a small number of studies but with a moderate number of participants as shown in Fig. 1. Although contact with authors may have led to further information being obtained. However, as prospective RCT registrations were all searched and update searches were carried out before publication, it is unlikely that a worthwhile completed study has been missed. It may appear on a cursory glance as if we focused on progesterone only without dydrogesterone, but it is important to highlight that studies were excluded based on route comparison strictly and reasons for exclusion were transparently given in Appendix 1. Regarding the quality of the studies, there was no blinding and performance bias remains a concern with respect to Domain 2 of the risk of bias assessment as there is no protection against preferential application of co-interventions to affect the outcome (Table 2, Fig. 2). However, lack of blinding may not introduce measurement bias as outcomes were objective. One limitation of studies included in this review is that there is no agreed protocol for progesterone supplementation in artificial ET cycles during FET at the international level. This meant that different doses of progesterone were compared in the evidence collated, leading to heterogeneity. This lack of consensus leaves the evidence synthesis and interpretation somewhat open, generating issues in conduct of meta-analysis and in generalizability of our findings for practice. The doses of progesterone were different in the various studies making meta-analysis unfeasible on account of heterogeneity in the comparisons available. With respect to this observed heterogeneity, it is possible that local clinician preferences have influenced the choice of interventions deployed in trials. This type of heterogeneity may be unavoidable at the current time, and future research on progesterone dosing regimens and schedules with robust evidence will help firm up unanimity in the field. This review showed that the overall evidence strength was low considering its various features (Table 5). Considering this uncertainty in the evidence, it is not possible to accept that the null hypothesis, i.e., there is no difference between routes, is true. The review merits consideration as the best available evidence synthesis at the time of writing.

Previous reviews of progesterone administration for LPS were either low to moderate in quality, did not cover all relevant administration routes or did not focus exclusively on artificial EP cycles [19–22]. Therefore, this is the most current, comprehensive, and focused review. The results of the evidence synthesis are in accordance generally with the previously published narrative reviews in that it is difficult to give guidance about the best practice at present given the low overall evidence quality. It is accepted that progesterone is a valuable intervention as its levels across luteal phase days are associated with pregnancy outcome in artificial FET cycles [30] and it also plays a role in prevention of miscarriage [10]. It remains to be seen why oral progesterone, while showing benefit in fresh cycles, is not beneficial in FET [31]. The role of low serum progesterone on the day of embryo transfer and use of multiple supplementation routes needs evaluation in artificial endometrial preparation [30, 32, 33]. The review’s findings serve as a scoping review in that it provides an overview of the key issues in the literature at present. They underpin the need for further high-quality multi-center trials in the future. The design of such trials would need to consider patient preferences in addition to those of the clinician. Ideally, the studies would need to be powered to detect differences in live birth rates. Future systematic reviews should undertake network meta-analysis comparing the outcomes in the various routes combining direct and indirect evidence to determine the rank order of the most effective option [34]. Even though it is well known that the vaginal administration of drugs is associated with inconvenience (vaginal irritation, discharge and bleeding) and that the oral route is non-invasive and less cumbersome, more patient preference data should be collected in future research. For full integration of this evidence in practice, patient acceptability and cost effectiveness studies will also be required.

In conclusion, the findings showed that there is little consensus and evidence is heterogeneous. The comparison of route of administration of progesterone for LPS during FET in artificial EP cycles needs more trials in the future.

## Data Availability

Not applicable.

## Appendix

See Table 6.

**Table 6:** Studies excluded with reasons from the systematic review of progesterone supplementation for luteal phase support

No	Author	Reason for exclusion*
1	(Tournaye et al. 2017)	P = fresh embryo transfer
2	(Salehpour, Tamimi and Saharkhiz, 2013)	P = undergoing controlled ovarian stimulation for IVF treatment (fresh cycle)
3	(Shiba et al. 2021)	I = 4 different types of vaginal progesterone
4	(Hershko Klement et al. 2018)	O = a difference in the number of sub-endometrial waves per minute with vaginal vs. IM progesterone
5	(Shiba et al. 2020)	I = 4 different types of vaginal progesterone
6	(Griesinger et al. 2018)	P = fresh cycle IVF
7	(Saharkhiz et al. 2016)	P = fresh intracytoplasmic sperm injection-embryo transfer cycles
8	(Ganesh et al. 2011)	P = undergoing controlled ovarian stimulation for IVF treatment (fresh cycle)
9	(Chakravarty et al. 2005)	P = women underwent IVF/(ICSI) treatment (fresh cycles)
10	(Chi et al. 2019)	P = fresh cycle
11	(Smitz et al. 1992)	I = intramuscular or intravaginal administration of natural progesterone in combination with oestradiol valerate for luteal phase supplementation in GnRHa cycles (Smitz et al. 1992)
12	(Moini et al. 2011)	P = ET was performed at the two to four cell stages, 40–44 h after insemination
13	(Beltsos et al. 2014)	P = fresh IVF cycles
14	(Dal Prato et al. 2008)	P = 2 days after oocyte retrieval, two embryos were replaced in the uterine cavity via the transcervical route
15	(Unfer et al. 2004)	P = the embryo transfer was performed at the 2- to 4-cell stage, 40–44 h after insemination
16	(Yanushpolsky et al. 2010)	P = fresh embryo transfer
17	(Zargar, Saadati and Ejtahed, 2016)	P = fresh embryo transfer
18	(Propst et al. 2001)	P = patients undergoing cryopreserved embryo transfers were excluded
19	(Tomic et al. 2015)	P = FET excluded
20	(Jiang et al. 2019)	Not an RCT
21	(Asoglu et al. 2019)	Not an RCT
22	(Venturella et al. 2018)	Not an RCT
23	(Fernandez-Sanchez, Gosalvez-Vega and Ninchritz, 2016)	Only an abstract
24	(Jiang et al. 2019)	Not an RCT
25	(Patki and Pawar, 2007)	P = with and without ovarian hyperstimulation syndrome
26	(Ozer et al. 2021)	P = modified natural cycle
27	(Bjuresten et al. 2011)	P = modified natural cycle
28	(Horowitz et al. 2020)	P = modified natural cycle
29	(Eftekhar, Rahsepar and Rahmani, 2013)	P = modified natural cycle
30	(Voung et al. 2021)	not an RCT
31	(Yuksel et al. 2021)	P = modified natural cycle
32	(Devine et al. 2018)	Interim report of full RCT included

*Reason for exclusion marked with letters related to PICO (Participants, Interventions, Comparisons and Outcomes) question; see Fig. 1 for flow chart of study selection
